# HIV epidemiology and responses among men who have sex with men and transgender individuals in China: a scoping review

**DOI:** 10.1186/s12879-016-1904-5

**Published:** 2016-10-20

**Authors:** Songyuan Tang, Weiming Tang, Kathrine Meyers, Polin Chan, Zhongdan Chen, Joseph D. Tucker

**Affiliations:** 1 University of North Carolina Project-China, No. 2 Lujing Road, Guangzhou, 510095 China; 2 Guangdong Provincial Center for Skin Diseases and STI Control, Guangzhou, China; 3 SESH Global, Guangzhou, China; 4 Kunming Medical University, Kunming, China; 5 Aaron Diamond Aids Research Center, New York, USA; 6 WHO China Office, Beijing, China; 7 UNAIDS China Office, Beijing, China

**Keywords:** HIV epidemics and responses, Men who have sex with men, HIV care continuum, Scoping review, China

## Abstract

**Background:**

Despite global efforts to control HIV among key populations, new infections among men who have sex with men (MSM) and transgender (TG) individuals are still increasing. The increasing HIV epidemic among MSM/TG in China indicates that more effective services are urgently needed. However, policymakers and program managers must have a clear understanding of MSM/TG sexual health in China to improve service delivery. To meet this need, we undertook a scoping review to summarize HIV epidemiology and responses among MSM and TG individuals in China.

**Methods:**

We searched MEDLINE, EMBASE and the Cochrane Library for recent studies on MSM/TG HIV epidemiology and responses. We also included supplemental articles, grey literature, government reports, policy documents, and best practice guidelines.

**Results:**

Overall, HIV prevalence among Chinese MSM was approximately 8 % in 2015 with a higher prevalence observed in Southwest China. TG are not captured in national HIV, STD, or other sexual health surveillance systems. There is limited data sharing between the public health authorities and community-based organizations (CBOs). Like other low and middle income countries, China is challenged by low rates of HIV testing, linkage, and retention. Several pilot interventions have been shown to be effective to increase HIV testing among MSM and TG individuals, but have not been widely scaled up. Data from two randomized controlled trials suggests that crowdsourcing contests can increase HIV testing, creating demand for services while engaging communities.

**Conclusion:**

Improving HIV surveillance and expanding HIV interventions for Chinese MSM and TG individuals are essential. Further implementation research is needed to ensure high-quality HIV services for MSM and TG individuals in China.

**Electronic supplementary material:**

The online version of this article (doi:10.1186/s12879-016-1904-5) contains supplementary material, which is available to authorized users.

## Background

In 2015 the WHO launched a comprehensive revision of the 2013 consolidated ARV guidelines, recommending immediate treatment for all people living with HIV and pre-exposure prophylaxis (PrEP) for people at high risk of HIV infection [1, 2]. At the same time, UNAIDS declared that the world should end the AIDS epidemic by 2030, with the goal of achieving the 90-90-90 diagnosis and treatment targets by 2020 [3]. Given these goals, it is imperative that we have a better understanding of key populations, including men who have sex with men (MSM) and transgender individuals (TG).

Globally, MSM are 19 times more likely to be living with HIV than the general population [4]. Despite global efforts to control HIV among key populations, new infections among MSM are still increasing [5–7]. High HIV prevalence was consistently observed among MSM in many regions around the world. For example in 2012, the HIV prevalence among MSM in the Caribbean was as high as 25 % [8]. Similar patterns have also been observed in Asia [8]. TG individuals are also an important, but often neglected, key population [9, 10].

The Chinese government has increased policy attention to HIV in recent years, providing an opportunity to enhance service delivery for MSM and TG individuals [11]. The purpose of this scoping review was to summarize HIV epidemiology and responses among MSM and TG individuals in China.

## Methods

We undertook a scoping review to summarize the MSM/TG HIV epidemics in China, sexual health services across the HIV care continuum, and interventions for Chinese MSM/TG. In addition, based on the literature review, we summarized several policy points for consideration.

We used Arksey and O’Malley’s framework for conducting this scoping study. Scoping studies summarize key evidence on a topic, but do not go through the process of a formal systematic review [12]. We identified studies published between January 2009 and October 2015 that reported on Chinese MSM HIV epidemiology, sexual health services across the HIV care continuum, and sexual health interventions. Studies were identified using keyword searches in electronic databases. We searched the following databases: MEDLINE (OVID interface, 1946 onwards), EMBASE (OVID interface, 1947 onwards) and the Cochrane Library. The search string used synonyms and variations of the following terms: MSM, TG, meta-analysis, review and China.

We included studies that had the following elements: 1) Study designs were systematic reviews; 2) Study participants were MSM or TG in China; and 3) Outcomes included data on health services across the HIV care continuum or interventions targeting Chinese MSM or TG. If data of interest were not available in systematic reviews, supplemental articles, grey literature, government reports, policy documents, and best practice guidelines were also included. We also contacted experts at the Chinese CDC, WHO, Gates Foundation and UNAIDS to provide reports, policy documents, and guidelines. Our preliminary report was reviewed by civil society organizations, UN organizations, the WHO China office, and two external reviewers.

## Results

### HIV epidemiology and surveillance

#### Population size estimation and data sharing

The WHO estimated 2–10 million MSM in China in 2009 [13]. China reported that about 2–4 % adult male population in urban areas and 1–2 % adult male population in rural areas are MSM, and estimated that the number of MSM in China may range from 3.1 to 6.3 million in 2009 [14]. Another study in China estimated that there were 5–10 million MSM in China [15].

Collected data is shared among some public health organizations and community-based organizations (CBOs). However, most of the MSM CBOs that work closely with local CDC have limited access to MSM health surveillance and related data [16–18].

#### HIV prevalence among Chinese MSM

**Fig. 1:**
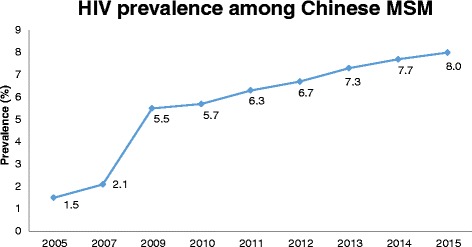
HIV prevalence among Chinese MSM based on Chinese surveillance system. Source: Chinese CDC [19] and AIDSInfo (http://www.aidsinfoonline.org/)

The HIV prevalence was estimated to be 6.0 % in 2010 [16], and it has gradually increased to 8.0 % in 2015 (Fig. 1) [19]. Regional disparity is an important feature of the HIV epidemic among Chinese MSM. Based on a systematic review, HIV prevalence is increasing across all regions of China, particularly in the southwest region, made up of five provinces [16]. The average HIV prevalence in this region was three times the overall Chinese average. In addition, municipalities and provincial capitals also have higher HIV prevalence, compared to other cities [20].

Young MSM worldwide and in Asia have increased risks of acquiring HIV and STDs [21]. In China, a study conducted across four cities reported an HIV prevalence of 6.7 % among MSM between 16 and 24 years old [22].

Routine HIV surveillance in China does not capture TG individuals and most of the studies reviewed did not mention TG individuals. One study found that 11.1 % of transgender individuals self-reported HIV infection [23] and another found a high burden of intimate partner violence and risky sexual behaviors [24].

#### Condom use and drug use among Chinese MSM

The results from six reviews [25–30] that summarized condom usage among Chinese MSM reveal that though large efforts (e.g., condom promotion in venues, peer education, and condom promotion through social media) have been made towards containing HIV, the overall rate of condomless anal intercourse (CAI) is still high. According to one meta-analysis that included 64 articles reported 82 studies [28], the CAI (during last 6 months) rate among Chinese MSM is about 54 % (95 % CI: 51–56 %). Data from the Chinese surveillance system showed that about 51 % of MSM reported engaging in CAI in the last 6 months in 2013 [19]. In addition to CAI with men, many Chinese MSM also have condomless sex with women. One meta-analysis that included 43 studies reported that the pooled estimates condomless sex rate with female sex partners was 74 % (95 % CI: 72–77 %) in the last 6 months [31].

A systematic review of 33 studies reported that the drug use rate ranged between 0.1 and 44 % among Chinese MSM (median = 2.4 %) [27]. In China, recreational drug use has increased considerably among MSM in recent years [30]. A multisite cross-sectional study reported that 28 % of MSM had used at least one kind of recreational drug in the last 6 months [32]. Among MSM, the most prevalent stimulant drugs were ecstasy and crystal meth. Popular non-stimulant drugs include Viagra and Ketamine [33].

### HIV care continuum

Since 2008, the China MOH carried out comprehensive AIDS responses with MSM populations in 61 cities across China, including expanding MSM surveillance, increasing HIV testing, case finding, and promoting linkage to care [20]. These policies helped to define and strengthen the HIV care continuum for MSM. Programs for HIV testing, linkage to care, adherence, and retention have all expanded in the past 5 years.

#### HIV testing

Data from national HIV surveillance system illustrated that the HIV testing rate (tested in last year) among Chinese MSM ranged between 43.2 and 49.0 % [19]. In addition, results from a meta-analysis of 18 studies suggested that only 47 % of Chinese MSM had ever tested for HIV, and 38 % had tested during the past 12 months [34]. The number of surveys among young MSM indicated that HIV testing rates in this group were less than 30 % [35]. In addition, among MSM tested for HIV, several studies suggested that HIV testing is infrequent [35–37]. One study noted poor HIV test uptake among TG individuals in China [23].

Several research studies have identified barriers and facilitators of HIV testing among MSM. Fear of stigma and discrimination, concerns about confidentiality, limited HIV knowledge, and institutional barriers such as inconvenient testing times were identified as barriers to accessing HIV testing [17, 38–40]. Practicing high risk sexual behaviors, family and partner support, rapid testing, and self-testing were mentioned as facilitators of testing [17, 38–40].

#### HIV linkage to care, ARV adherence and retention in care

Nationwide scale-up of HIV treatment programs has been implemented in China over the last decade. However, late diagnosis, incomplete linkage to care, and high rates of loss-to-follow-up remain major clinical and public health challenges in China [41]. Infrequent viral load monitoring and incomplete reporting within surveillance systems complicate more detailed analyses. Further strengthening of the surveillance system and implementation research are needed. A study based on the national epidemiology database reported that during 2006–2012, 21.3 % of newly diagnosed MSM did not receive CD4 cell count testing within the 6-months following diagnosis [42]. A online survey study reported consistent results (74.6 % of positive individuals saw a doctor in 6 months and 25.4 % took over 6 months to be linked to care) [43]. Multistage stepwise HIV testing and treatment initiation procedures are major structural barriers to providing timely antiretroviral therapy [41].

A study conducted in 2014 indicated that many HIV-infected individuals are receiving ART that is sub-optimal, even after they receive definite diagnosed [44]. Among MSM patients with CD4 cell count ≤ 350 cells//mm3, only around 60 % received ART [44, 45]. A study conducted in Guangzhou reported that among 721 adults living with HIV receiving ART, 18.9 % reported recent non-adherence (any missed ART in the past 4 weeks) and 6.8 % reported treatment interruption (4 or more weeks of missed ART in the past year) [46].

Retention in care among MSM patients is also not optimal [41, 42, 44]. Based on a study conducted during 2003 to 2009, people living with HIV receiving ART reported that the median length of time on ART was 18.9 months, and the cumulative probability of attrition from ART initiation was 9 % at 12 months and 24 % at 60 months. This nationwide study showed that MSM retention rates were better than other risk groups. A retrospective cohort study conducted in Changsha reported a retention rate of 58.1 % at 12 months, with no difference in retention reported between MSM and others [47].

These studies also showed that MSM face a number of individual and structural barriers across the HIV care continuum. Negative coping methods create significant problems for linking newly-diagnosed men to care and retaining them in care. Meanwhile, qualitative research indicates that nondisclosure of sexual orientation to families, absence peer social support and professional psychological counseling, lack of affordable and specialized treatment and care, homophobia and discrimination from providers may deter engagement in care [40, 48].

### Interventions ongoing and completed

#### Prevention and behavioral interventions at individual and group-level

Since 2005, the Chinese government has expanded its intervention efforts to MSM [49]. Various programs have been conducted to promote condom use, counseling and testing, peer education, and follow-up outreach. A meta-analysis that summarized 22 MSM intervention studies suggested that behavioral interventions have been efficacious in increasing HIV knowledge, HIV testing, and condom use, and in reducing sexual risk behaviors. However, the study also indicated that interventions have not reduced the incidence of HIV among Chinese MSM [50].

#### Interventions for promoting HIV Self-testing (HIVST) in China

HIVST is not illegal in China, and more than 20 HIVST kits have been approved by the Chinese Food and Drug Administration [51] and are available in China [52]. There is currently no national program of HIVST among MSM in China, however around one quarter of Chinese MSM have self-tested for HIV [52, 53]. Several pilot programs have shown that HIVST is an additional HIV testing method that could increase first-time HIV testing [53, 54]. Many pilot interventions have been conducted to examine the efficacy of hybrid CBO-clinic models in promoting HIV testing and linkage to care (Table 1). These interventions primarily focus on improving cooperation among CDC system, hospital and MSM CBOs in China [46, 55]. For example, by collaborating with a local CBO, the Guangzhou CDC built a social entrepreneurship model to promote HIVST among MSM [56]. Overall, 198 (52.1 %) MSM purchased self-testing kits, and 192 (97.0 %) participants used kits and returned the testing results [57].

**Table 1:** Project IMPACT: A Model for Increasing the Collaboration between Community-based Organizations and the Public Sector in China

Purpose of the program
A pilot project “Integration Minimum package of Prevention in Accelerating Case finding and Treatment (IMPACT) was carried out in Guangzhou since 2008. The purpose of this project was to increase the collaboration between Community-based Organizations (CBOs) and the public sector in China to provide friendly HIV-related service to men who have sex with men (MSM).
Methods of the program
Guangzhou CDC and the Lingnan Partners Community Support Center worked together to design an integrated service including HIV health education, online HIV risk assessment, on-site HIV counseling and testing, partner notification, CD4 cell count testing, psychosocial care and support, and guidance on clinical treatment. This program includes three main parts: online prevention tools, online-to-offline service linkage, and one-stop service.
*Online prevention tools:* Two internet tools were developed: a scenario-based application and an HIV risk self-assessment system. The scenario-based application is an interactive internet application that simulates real-life HIV risk scenarios, in order to promote HIV testing and condom use. The online HIV risk self-assessment system evaluates an individualized HIV-risk score by evaluating an individual’s risk profile. Based on the results, the system provides tailored guidance to promote HIV testing and behavioral change.
*Online-to-offline service linkage:* This component linked virtual intervention/testing mobilization efforts to actual HIV testing, and facilitated HIV care. The online prevention tools described above were linked to an online appointment system for HIV testing. Test results were made available online by an anonymous fication system (Easy Tell®)
*One-stop shops:* In ‘one-stop shops’, public sector staff provided on-site blood sampling and testing and carried out epidemiological investigations. Meanwhile, CBO peers delivered high-quality and timely pre- and post-test counseling, psychosocial support services, guidance on retention to care and ART adherence support services.
Outcomes
The IMPACT model provides services on HIV health education, online HIV risk assessment, on-site HIV counseling and testing, partner notification, CD4 cell count testing, psychosocial care and support, and ART adherence guidance. The number of tests performed through IMPACT has increased from 1,064 in 2008 to 7,754 in 2013. Of the 999 HIV positive cases identified through this project between 2008 and 2013, linkage to care and retention in care rates of 95 and 89 %, respectively [58].
Lessons learned
This public sector-CBO hybrid model has not only addressed the needs of the MSM community, but has also been instrumental in reducing barriers of access to HIV services.

#### Internet-based and social media interventions

A few internet-based interventions have been conducted to improve accessibility to HIV services and care [55, 58, 59]. For example, the Guangzhou CDC developed two internet-based interventions: ‘scenario experiencing intervention application’ and ‘online HIV risk self-assessment system’ [58]. Compared to the control group, participants in the intervention arm of HIV risk-assessment system had reduced CAI in the last anal sex by 44 %, and CAI with regular and casual partners within the last three months by 26 and 25 % respectively [58].

Various social media techniques have been applied to HIV educational interventions as well as to testing mobilization and partner services interventions targeted towards MSM in China. Danlan Gongyi, an LGBT online web portal and CBO, used Wechat to provide rapid and anonymous HIV testing services as well as offline services such as psychological counseling and support [3]. Other social media platforms, such as QQ, Jack’d and microblogging, have been also used in carrying out HIV/AIDS interventions among Chinese MSM [60, 61].

#### Crowdsourcing HIV test promotion interventions

Crowdsourcing is “the practice of obtaining needed services, ideas, or content by soliciting contributions from a large group of people and especially from the online community [62].” Crowdsourcing can be used to solicit concepts, images, and videos to promote HIV testing (Table 2). Several crowdsourcing contests have been implemented in South China. One randomized controlled trial (RCT) in China aimed at promoting HIV testing showed that crowdsourcing methods were both effective and cost saving when compared to a health marketing intervention. HIV test uptake was similar between the crowdsourced arm (37 %, 114/307) and the health marketing arm (35 %, 111/317) [52]. Another RCT in China found that a crowdsourced intervention was non-equivalent to a social marketing intervention, also noting cost savings [63].

**Table 2:** SESH and crowdsourcing contests to create demand for HIV testing and other services

Purpose of the Program
To create more engaging and effective sexual health services using crowdsourcing and other social entrepreneurship tools.
Methods of the program
SESH has organized and evaluated several creative contributory contests and other programs [76]. For example, in 2013, we launched our first contest to solicit videos promoting HIV testing. First, SESH posted an online open call for videos and hosted a call to increase awareness of the contest. Second, a group of multisectoral judges evaluated each of the video entries, giving them a score of 1–10 and selecting a group of finalists and a single winner. Judging criteria included reaching individuals who had never tested before, generating excitement, and community responsiveness. Finally, the winner from the contest was formally evaluated and selected as an intervention tool.
In 2014, SESH launched a sexual health image contest to encourage young students to talk about sexual health. We promoted the contest information as well as sexual health knowledge among young people though online (broadcasting and interacting on wechat and weibo) and in-person events (lectures, activities, and workshops). In 2015, SESH launched a condom video contest to solicit videos promoting condom use among MSM. Then in 2016 the World Health Organization invited SESH to organize a global contest to solicit hepatitis testing innovations [73].
Outcomes
SESH has data from RCTs, qualitative research, and social media suggesting the effectiveness of crowdsourcing as an approach. Two RCTs demonstrated that crowdsourcing was effective and saved money compared to conventional evidence-based marketing approaches [52, 63].
Lesson learned
Crowdsourcing could be a useful way to spur creative, new ideas for improving health and engaging communities. This new tool may be especially useful in low and middle-income countries where civil society organizations are often constrained or less able to directly inform public health programs. Crowdsourcing contests may help create more engaging, effective, and creative campaigns [33, 35].

#### Interventions for improving linkage and retention in care

In 2012, the China CDC initiated a pilot intervention project to demonstrate a ‘one-stop’ service model for individuals with HIV infection [44]. This model included referral, transportation, information and emotional support. Patients in the service clinic receive all procedures and protocols in a single setting. The pilot intervention demonstrated that this model outperformed the existing service model on five out of six indicators. Between 2012 and 2014, the Chinese CDC in Guangxi also piloted a program for immediate initiation of ART [41]. The intervention significantly increased ART coverage within 90 days of diagnosis of HIV infection, and reduced overall mortality from about 26 % to less than 10 % [41].

#### Pre-exposure prophylaxis (PrEP)

The use of PrEP is now recommended by the WHO for all individuals at substantial risk of acquiring HIV [64]. China has yet to make new recommendations with regard to PrEP. Few studies suggest that while awareness remains low (ranging from 2.8 to 22 %), willingness to use PrEP is high (ranging from 64.0 to 84.6 %) [65–68].

Two PrEP demonstration projects in China have been published to date [69, 70]. The first one compared the effect of combination regimen of TDF + FTC daily to TDF + FTC taken twice weekly plus an additional dose 2 h before sex. Among 153 MSM, 79.7 % completed 28 weeks of follow up, and 68.6 % reported not missing a dose [69]. The second study allowed participants to choose the no drug intervention, daily TDF or peri-coital dosing (1 dose TDF 24–48 h before sex plus 1 dose TDF within 2 h after sex). Retention at 12 months was relatively low (43.1 %), and varied significantly across study arms [70].

## Discussion

Based on the epidemiology and response data, we identified a number of opportunities to strengthen the HIV response and decrease HIV risk among MSM in China. These opportunites include improving HIV surveillance and expanding interventions among MSM and transgender individuals.

### Improving HIV surveillance

Given the size and complexity of China, an accurate MSM/TG HIV surveillance system is critical. Two areas could improve HIV surveillance among these key populations: 1) enhancing data sharing between CBOs and CDCs; 2) using online surveys to capture MSM and TG individuals. Several interventions suggest the importance of collaborative CBO-CDC programs across the continuum of care. However, MSM CBOs often have limited access to MSM sexual health surveillance data. Increased data sharing between CBOs and local CDCs could improve service provision, needs assessment, and intervention development.

Online surveys can also help capture real-time HIV surveillance data. Large numbers of MSM in China routinely use the Internet [20], especially smart-phone based partner-seeking mobile applications [49]. Some online MSM are difficult to reach through the current MSM surveillance system [23]. Integrating online data collection into the surveillance system could make it easier to reach some subgroups of MSM. Currently, there is very limited data specific on TG individuals. Conducting online surveillance among online TG individuals could be useful, as previous studies have shown that online sampling can reduce some of the social and structural barriers that limit efforts to reach TG [23, 24]. Data security and confidentiality are important concerns in the context of using online surveys.

### Expanding HIV interventions for MSM and TG individuals

Our review identified a number of MSM/TG interventions that could be expanded, including the following: 1) HIVST interventions to expand HIV testing; 2) crowdsourcing and social media interventions to stimulate demand for HIV services; 3) community-based interventions.

HIVST may be a useful tool for expanding HIV testing and promoting people-centered systems. Although HIVST systems tailored for MSM have been piloted in many Chinese cities [53, 57, 58], none have been widely scaled up. Expanding the use of HIVST among MSM represents a major opportunity for public health systems to reach MSM and TG individuals with suboptimal HIV test uptake. The expansion of HIVST may require new organizations such as a social enterprise [71] which combine elements of enterprise and social welfare organization.

Both crowdsourcing and social media interventions have proven effective in stimulating demand for HIV services in China. Two randomized controlled trial studies demonstrated that a crowdsourcing approach was effective and cost saving method among MSM and TG individuals in China [52, 63]. This method could increase community engagement in HIV campaigns [52]. A crowdsourcing approach could also be used to increase demand for PrEP and partner services. In addition to crowdsourcing, other types of social media interventions may help to increase HIV service demand [72, 73].

Community-based interventions are an important part of the HIV response among MSM and TG individuals. Supporting local community-based organizations to implement community-based interventions should be considered. At a national and sub-national level, the participation of key populations and key populations living with HIV must be considered in formulating strategies and programming [58]. Within the many CDC-CBO hybrid models, ensuring governance and resources to support community voices is important.

## Conclusion

Implementation of HIV interventions, especially those focused on serving MSM and TG individuals, is an enormous challenge [74]. Implementation science research can help identify and anticipate problems, and in turn, answer questions on how best to deliver interventions [75]. Incomplete surveillance and reporting systems, especially for TG individuals, underlines the need for strengthened routine surveillance and further research. This scoping review suggests several unmet implementation needs for MSM and TG in China.

## Additional file

Additional file 1: Annex: Summary of ongoing and completed interventions focus on the HIV care continuum among MSM in China (DOCX 137 kb)

## Abbreviations

ART: Antiretroviral therapy
ARV: Antiretroviral(s)
CAI: Condomless anal intercourse
CBOs: Community based organization(s)
CDC: Centers for disease control and prevention
HIVST: HIV self-testing
HTS: HIV testing service
ISR: Implementation science research
KP: Key population(s)
LGBT: Lesbian, gay, bisexual and transgender
MSM: Men who have sex with men
PLHIV: People living with HIV
PrEP: Pre-exposure prophylaxis
PS: Partner services
RNA: Ribonucleic acid
STDs: Sexually transmitted diseases(s)
STIs: Sexually transmitted infection(s)
UNAIDS: Joint United Nations Programme on HIV/AIDS
VCT: Voluntary counselling and testing
VL: Viral load
WHO: World Health Organization
