# Supplementary material for: HIV epidemiology and responses among men who have sex with men and transgender individuals in China: a scoping review
Source: BMC Infect Dis. 2016 Oct 20;16:588. doi: 10.1186/s12879-016-1904-5 (PMC5073436; doi:10.1186/s12879-016-1904-5)
Supplement: Additional file 1: — Annex: Summary of ongoing and completed interventions focus on the HIV care continuum among MSM in China (DOCX 137 kb) [file 12879_2016_1904_MOESM1_ESM.docx]

Annex: Summary of ongoing and completed interventions focus on the HIV care continuum among MSM in China

| **Cascade step** | **Intervention Project** | **Focus / Principal interventions** | **Outcomes** | **Location (time period)** |
| --- | --- | --- | --- | --- |
| **Prevention and behavioral interventions** | Behavioral intervention project among MSM in Chongqing city | **Focus:** To assess the effectiveness of behavioral interventions among MSM and to provide suggestions for the development of intervention strategy for this population.  **Interventions:**  - Peer education  - Behavior Change Communication  - VCT  - Condom promotion  - Standard STDs treatment | - CWM-1: increased from 56.4% to 64.4%  -CWM-2: increased from 68.2% in 2006 to 63.3%.  HIV testing: increased from 18.9% to 35.2% | Chongqing  (2006-2008) |
|  | Feasibility of universal HIV testing among MSM | **Focus:** To understand the status of AIDS related knowledge awareness high risk behavior and sexual transmitted infections among MSM after intervention and evaluate the intervention effect.  **Interventions:**  -Three annonymous questionnaire survey were conducted before and 6,12months after intervention and blood samples were taken for HIV and syphilis detection among MSM. | -CWM-1: from 72.17% to 75.10%  -CWM-2: from 43.04% to 45.20%  -HIV testing: from 28.26% to 44.69%  -STD: The prevalence of syphilis was from 0.43% to 11.28%  -HIV: from 0.87% to 2.43% | Not report (2007.08/09-2008.09/11) |
|  | China - Merck Sharp & Dohme AIDS cooperation program: Community-based intervention project among MSM | **Focus:** To evaluate the effect of a community -based intervention project among MSM  **Interventions:**  - Promoting VCT through peer education and outreach activities  - One-to-one pretest counseling  - Linkage to care  -STD treatment | -HK: from 56.8% to 87.0%  -CWM-1: from 58.9% to 75.8%  -CWM-2: from 12.5% to 27.5%    -HT: from 44.4% to 65.2%. | Not report (2008.06-2010.06) |
| **Prevention** | A peer-driven behavioral  Intervention to reduce HIV-related risk among MSM | **Focus:** To test the feasibility of a peer-driven behavioral intervention and to evaluate efficacy in reducing HIV-related risk among men who have sex with men (MSM)  **Interventions:**  Peer-driven behavioral intervention（4 sessions） was chosen to influence the MSM peer networks   - Behavior labeling and evaluating individual high risk behaviors. - Developed individualized plan to make a commitment to changing their high-risk behaviors - Emphasized taking action to change high-risk behaviors - Addressed ways to deal with barriers to practicing safe sex | **CWM-1:** increased from 55.3% to 65.2%  - HIV testing: from 10.0% to 52.4%. | Anhui (2006.05-2006.10) |
| **Prevention** | Enhanced voluntary counseling and testing (EVCT) project to promote condom use among MSM | **Focus:** the relative efficacy of an enhanced (EVCT) versus standard (SVCT) voluntary counseling and testing in reducing unprotected anal intercourse (UAI) among men who have sex with men (MSM) in China  **Interventions:**  EVCT group watched a theory-based video narrated by a HIV positive MSM, received enhanced counseling and a reminder gift | -CAI(with any male sex partners): from 48.4% to 66.7%  -CAI(with regular male sex partners): from 52.2% to 68.9%  -CAI(with casual male sex partners): from 36.8% to 52.5% | Nanjing (2008-2009) |
|  | One-to-one QQ-based internet counseling project | **Focus:** Understand the effectiveness of intervention services among MSM through one-to-one QQ-based internet counseling  **Interventions:**  One-to-one QQ based internet counseling | -CWM-1: from 66.4% to 81.5%  -CWM-2: from 45.3% to 59.6%  -HIV Testing: from 57.3% to 68.3% | Harbin (2011.10-2012.12) |

CWM-1, condom use during the most recent sexual intercourse with a man ;

CWM-2,condom use during intercourse with a man during the past 6 months

CAI, condomless anal intercourse ; CWW, condom use during intercourse with a woman during the past 6 months）

| **Cascade step** | **Intervention Project** | **Focus / Principal interventions** | **Outcomes** | **Location (time period)** |
| --- | --- | --- | --- | --- |
| **Diagnosis and linkage to treatment/care** | The China-Gates Foundation HIV Prevention Cooperation Program | **Focus:** Collaboration between local CDC, hospitals, and CBO in order to expand testing, treatment, and prevention among Chinese populations most at-risk for HIV infection (MARPs), especially MSM.  **Interventions:**  - Emphasized a “3-in-1” operational structure with synergistic collaboration between three key partners:the government (CDC), hospitals, and CBOs.  - Prevention for MARPs–to increase the coverage and improve the quality of prevention interventions for IDUs, FSWs and MSM, in order to reduce high-risk behaviors and increase HIV testing.  - Prevention with positives–to expand and improve follow-up, support, and prevention interventions for PLHA to reduce further transmission of HIV infection. | -The number of MSM diagnosed as HIV-positive increased seven-fold within four years, from 646 in 2008 to 4,536 in 2012.  - The number of PLHA eligible for ART and who were actually on ART increased ten-fold, from 1,284 in 2008 to 12,850 in 2012.  - The number of pre-ART PLHA tested for CD4 increased seven-fold, from 3,576 in 2008 to 24,838 in 2012, and the CD4 test rate increased from 47.4% in 2008 to 84.4% in 2012.  -The follow-up rate on pre-ART HIV-positive individuals jumped from 42.5% in 2008 to 94.3% in 2012. | 14 urban cities and Hannan province (2007-2012) |

| **Cascade step** | **Intervention Project** | **Focus / Principal interventions** | **Outcomes** | **Location (time period)** |
| --- | --- | --- | --- | --- |
| **Diagnosis and linkage to treatment/care** | MSM peer-led, CBO-based, mobile rapid HIV testing program | **Focus:** Outcomes of a peer-led, community-based intervention providing rapid HIV testing and case management for linkage to care for untested MSM.  **Interventions:**  - Provinial CDC + MSM CBOs  - Counselling with HIV rapid testing at gay-oriented venues  - CBOs refer MSM initially screened HIV positive to the local CDC for repeat and confirmatory testing  - Social support and case management up to the point of initiating their enrolment in HIV care | Achieved higher proportions of HIV- positive MSM screened by the CBO received their confirmatory test results (98.1% vs 72.6%) and linked to care (90.4% vs 42.0%), compared with those in the surveillance surveys. | 4 counties in Jiang Su Province (January to June 2012) |
|  | The Home-Based HIV Self-Testing Project in Beijing city | **Focus:** Feasibility and acceptability of home-based HIV self-testing among Chinese MSM  **Interventions:**  - HIV rapid test kit was shipped to participants  - Pre-test counseling via a telephone hotline or QQ Group  - Send picture of the testing strip via QQ or email to CBO staff  - Linked to a local CDC for a second screening test | - Home-based HIV self-testing is an alternative approach for increasing the coverage of HIV testing among Chinese MSM.  - CBO played an important role in the pilot project | Beijing (2012) |

| **Cascade step** | **Intervention Project** | **Focus / Principal interventions** | **Outcomes** | **Location (time period)** |
| --- | --- | --- | --- | --- |
| **Diagnosis and linkage to treatment/care** | Spurring innovation in HIV testing and linkage: a crowdsourcing approach (Quasi-experimental trial) | **Focus:** Effectiveness of a crowdsourced intervention and a social marketing intervention on HIV testing and linkage among young MSM  **Interventions:** Crowdsourced intervention and Social marketing intervention | Access to HIV testing, linkage to care, HIV transmission | Guangdong and Shangdong Provinces (2014-2019) |
|  | Structurally Simplified HIV Testing and Treatment pilot project in China | **Focus:**  Evaluate the effectiveness of the simplified test and treat intervention in reducing delays to treatment and decreasing mortality.  **Interventions:** Completed within a week of the first positive HIV screening test result, incorporating immediate HIV confirmatory testing, pre-ART CD4 testing, pretreatment counseling, and ART initiation regardless of CD4 count. | - Mortality decreased from 27% to 10% for all cases  -Receipt of baseline CD4 testing within 30 d of HIV confirmation increased from 67% to 98%  -The time from HIV confirmation to ART initiation decreased from 53 days to 5 days  - Initiation of ART increased from 27% to 89% | China (2012 onwards) |
|  | “One-stop service” Pilot intervention project (integrating a range of public health resources within a ‘‘one-stop’’ service delivery protocol) | **Focus:** Describe the treatment experience, follow-up participation and disease progression among patients who enroll in One-stop service delivery model of HIV/AIDS care.  **Interventions:**  The new clinic included counseling, testing, diagnosis, treatment and health education provided by physicians, nurses and workers from community-based organizations (CBOs) serving MSM. The ‘‘one-stop’’ services included cART provision, regular follow- up, referral for Tuberculosis (TB) screening and treatment, referral for AIDS- related or non-AIDS-related diseases, risk behavior prevention, and psychological support. | -Proportion receiving tests for CD4 cell count at the six-month interval was 98.2%  -Proportion with HIV suppression for patients receiving cART for 6 months was 86.5%  -Proportion with CD4 cell recovery for patients receiving cART for 12 months was 55.8% - - Median time from HIV confirmation to first test for CD4 cell count was 7 days  - Median time from first CD4 cell count 350 cells/mL to cART initiation was 26 days | Wuhan (2011-2013) |
